# A Potential Multimodal Test for Clinical Assessment of Visual Attention in Neurological Disorders

**DOI:** 10.1177/15500594221129962

**Published:** 2022-10-03

**Authors:** Valentina Barone, Johannes P. van Dijk, Mariette H.J.A. Debeij-van Hall, Michel J.A.M. van Putten

**Affiliations:** 1 Clinical Neurophysiology (CNPH), TechMed Centre, 3230 University of Twente, Enschede, Netherlands; 2 Twente Medical System International B.V. (TMSi), Oldenzaal, Netherlands; 3 3804 Academic Center for Epileptology Kempenhaeghe, Heeze, Netherlands; 4 Eindhoven University of Technology, Eindhoven, Netherlands; 5 Department of Clinical Neurophysiology, Medisch Spectrum Twente, Enschede, Netherlands

**Keywords:** visual attention, cognitive task, clinical neurophysiology, EEG, eye-tracking

## Abstract

Attention is an important aspect of human brain function and often affected in neurological disorders. Objective assessment of attention may assist in patient care, both for diagnostics and prognostication. We present a compact test using a combination of a choice reaction time task, eye-tracking and EEG for assessment of visual attention in the clinic. The system quantifies reaction time, parameters of eye movements (i.e. saccade metrics and fixations) and event related potentials (ERPs) in a single and fast (15 min) experimental design. We present pilot data from controls, patients with mild traumatic brain injury and epilepsy, to illustrate its potential use in assessing attention in neurological patients. Reaction times and eye metrics such as fixation duration, saccade duration and latency show significant differences (p < .05) between neurological patients and controls. Late ERP components (200–800 ms) can be detected in the central line channels for all subjects, but no significant group differences could be found in the peak latencies and mean amplitudes. Our system has potential to assess key features of visual attention in the clinic. Pilot data show significant differences in reaction times and eye metrics between controls and patients, illustrating its promising use for diagnostics and prognostication.

## Introduction

Attention, the cognitive process of selectively concentrating on a particular task, is an essential function of the human brain.^1^ Attention requires filtering and subsequent processing of external and internal stimuli, which is critical in e.g. learning and responding properly to relevant inputs. Attention is also an essential component of our interactions with the environment, ranging from social relationships to cycling or driving a car.^2^

In various neurological conditions, attention is affected. Examples include neurodegenerative disorders, stroke, epilepsy^3^ and traumatic brain injury (TBI).^4^ Attention deficits may warrant limitations of every-day activities to protect patients and their environment e.g. to prevent falls or reduce risks associated with driving a car. This comes at a cost: impediments of movements and work-related activities, social isolation and consequent deficits in social interactions are commonly observed in patients with epilepsy and TBI.^5,6^ In these and other neurological conditions, limitations and risks differ and personalized advice is crucial.^7^ For instance, in some children with absence epilepsy, everyday activities such as swimming or bicycle riding may be dangerous,^8^ but this is not the case in patients with elementary seizures that manifest as olfactory sensations, only.

Risk assessment is typically based on common sense or experience of the treating physician. A way of assisting in more objective clinical advice is providing objective measures reflecting critical elements of every-day activities, which depend on attention and its subcomponents.^9^ Here, we focus on the assessment of reaction time (RT), oculomotor metrics, and electroencephalographic (EEG) derivatives i.e. ERPs, all acknowledged markers of the attention system.^10^

In patients with epilepsy and mild TBI (i.e. Glasgow Coma Scale: 13–15, loss of consciousness *<* 30 min and/or post-traumatic amnesia < 24 h^11^), our clinical population of primary interest, several studies have reported changes in attention assessed with these markers. For instance, RT is longer in children with genetic generalized epilepsy.^12,13^ Similar results have been reported in patients with mild TBI, both within 24 h and one month after the injury.^14–16^ Oculomotor functions are known to be linked to perceptual visual attention, too.^17^ Patients with mild-to-severe TBI tend to spend less time on visual target stimuli compared to controls.^18^ In the same population, other eye-tracking metrics reveal possible dysfunctions of the visuo-attentive system, reflected in larger position errors, smaller saccadic amplitudes, smaller peak velocities, smaller peak accelerations and longer saccadic durations, that may improve after the acute phase.^19,20^ Patients with epilepsy may show shorter fixation duration on visual target stimuli,^21^ upward eye deviation,^22^ increased saccadic peak velocity, reduced latency of prosaccades and increased express saccades.^23^ ERPs have been largely employed to study cognitive features and deficits of attention.^24^ Among others, the P300 component, visible around 250–450 ms after stimulus presentation, is involved in high-level cognitive abilities, such as attention.^25^ The latency of P300 in central line channels is longer than healthy controls in patients with genetic generalized epilepsy (*≥* 20–30 ms)^26^ and in TBI (*≥* 80 ms) both in the acute (≤ 24 h) and sub-acute (48 h up to 3 months) phase,^27^ while it is similar to controls in the chronic phase of TBI (*≥* 3 months).^15^ The P300 mean amplitude is also affected: in chronic TBI patients, the mean amplitude is smaller than in controls^15^; similar findings were reported in epileptic patients.^28^ Usually, latency and mean amplitude of P300 correlate positively with manual RT,^29,30^ and a significant difference in RT may result in relatively discrepant ERP components to standard values.

The integration of these known metrics (i.e. quantitative EEG,^31^ eye-tracking^32^ or subcomponents of RT^33^) in clinical practice can provide an objective and reliable assessment of the attentional status of neurological patients.

We present an integrated test to possibly assess metrics of visual attention in a single experimental design, employing a computerized choice reaction time (CRT) task, a screen-based eye tracker and an EEG. Such multimodal test may represent an innovative way of objectively measuring attention in the clinic. We report the technical details of our system and present preliminary results obtained from pilot measurements in healthy controls and patients with epilepsy and TBI. We address the following research questions:

(1) is our setup suitable to measure parameters of visual attention in a clinical setting and (2) has our test potential to discriminate between healthy subjects and patients with neurological disorders?

## Materials and Methods

### Stimuli and Apparatus

**Figure 1.**
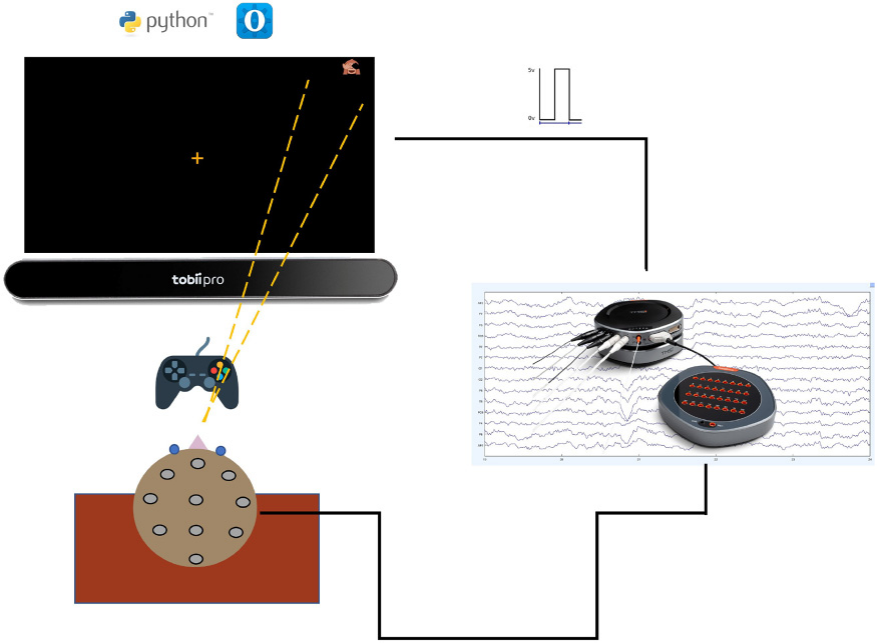
Schematic of the measurement system. The subject is seated in front of the laptop, where the CRT task is presented. The ET is positioned below the screen. The subject interacts with the task with a gaming controller. Meanwhile, EEG is recorded. A TTL trigger signal is sent to the EEG data every time a target stimulus appears on the screen. More details about the setup can be found in section S1 and S2 of the Supplemental Material.

A schematic representation of our portable setup is shown in Figure 1. A computerized CRT task for visual attention was developed using Python 3.7.6 with the open-source package OpenSesame (OS) 3.3.9,^34^ using the PsychoPy back-end. The task consists of responding to a particular stimulus with a game controller (pressing a left or right sided button with both hands). The sequence with relative timings and stimuli sizes are shown in Fig. S1 of the Supplemental Material. Accuracy values (right or wrong button press), single trial and mean RTs for each condition are obtained from the task. On the bottom of the laptop screen, a screen-based eye-tracker (Tobii Pro Nano, Tobii Technology, Danderyd, Sweden) is positioned to detect eye movements. The eye-tracker has a sampling frequency of 60 Hz, accuracy of 0.3° in optimal conditions and precision of 0.10° RMS in optimal conditions. From the eye-tracking data we extract saccade parameters (i.e. amplitude, velocity, duration) and fixation durations (for more details on eye tracking metrics, please refer to section S3 of our Supplemental Material). Further, during the tasks an EEG is recorded to extract ERPs.

### Procedure

The CRT task we developed mimics a simplified version of the Attention Network Task (ANT).^35^ This allows to easily test the alerting and executive networks of attention using a within-subject repeated-measure experimental design with 3 conditions: (A) no cue, (B) congruent cue and (C) incongruent cue. Condition (A) is met when no white dot precedes the target stimulus. A cue is congruent (B) if it lies in the same position on the screen as the subsequent target stimulus, while it is incongruent (C) when it lies in another position. Cue conditions are randomized during the task. Among the cued conditions, the position of the cue and of the target stimuli varies randomly among 8 positions. The participants are instructed to look at the sequence of objects appearing on the screen.

Whenever a target stimulus (i.e. monkey) appears, the subject has to press a button on a gaming controller only if one of the two hands on top of the monkey's eyes is black: the left button (LB) if the hand is on the left side of the screen, the right button (RB) if the hand is on the right side of the screen. If none of the hands are colored black, the subject should not press any button. Each participant performed 6 practice trials, followed by 112 experimental trials during which EEG and eye movements have been recorded. Details on the time synchronization of our equipment can be found in the Supplemental Material S2. In total, the measurement procedure takes approximately 15 min ( + 10 min preparation).

### Eye Movements

The output data from the ET consists of the x- and y-coordinates of the left and right eye, time stamps, and the pupil sizes of both eyes. The ET data was obtained after performing a fully automated five-point calibration procedure at the beginning of the task.

*Preprocessing of ET data* Eye movements analyses were performed with Python 3.8.3. Trials with mean RT below 200 ms and above 1700 ms were excluded from further analyses.^36^ Raw data was preprocessed following Tobii I-VT fixation filter algorithm.^37^ A linear interpolation was performed on small gaps,^38^ i.e. invalid values in the raw data shorter that 75 ms, caused by frequency correction of the eye tracker. A moving average filter was applied with a window of 150 ms (i.e. 9 samples) to smooth the raw data.

*Eye tracking features* We used a velocity-based algorithm (velocity threshold identification, I-VT) to extrapolate saccades and a dispersion-based algorithm (dispersion threshold identification, I-DT) to extrapolate fixations. More details about the two algorithms and the motivation of this choice can be found in section S3 of the Supplemental Material. As a first step, we defined two areas of interest (AOI): the fixation cross, *T*_X_ and the target stimulus, *T*_stim_. Fixations were determined using the pointwise dispersion of gaze points of the left and right eye averaged together. Only left or right eye was used if the corresponding opposite eye showed consistently *≥*100 pixels difference with the correct gaze position. Fixations were determined per trial, if both *T*_X_ and *T*_stim_ were visited. We defined a fixation if more than 10 consecutive gaze points (i.e. 160.6 ms^39^) were included in either *T*_X_ or *T*_stim_. The percentage of time spent in each AOI was calculated as well.

We further define saccadic amplitude, *S*_amp_ as

(1) $$S_{\mathrm{amp}}=D_{\mathrm{tf}}\frac{\frac{180}{\pi }\arctan\;(\frac{0.5h}{d})}{0.5r},$$

where *D*_tf_ is the distance in pixels between *T*_X_ and *T*_stim_, *h* is the height of the screen in cm, *r* the vertical resolution of the monitor in pixels and *d* is the distance from the screen in cm. Saccadic duration, *S*_Δt_ was defined as the time difference between the end and the beginning of the saccade, and the mean saccadic velocity, *versus* as

(2) $$v_{s}=\frac{S_{\mathrm{amp}}}{S_{\Delta t}}.$$

Furthermore, we derived the subcomponents of total RT. Saccadic latency (SL) was calculated as the sampling time of the first sample included in the saccade. Visual reaction time (VRT) equals our saccadic duration. Processing speed (PS) was found subtracting the sum of SL and VRT to the total RT. If one of the three RT components could not be detected we discarded the entire trial.

### EEG

EEGs were recorded with Ag/AgCl electrodes attached according to the 10–20 system. Different EEG amplifiers were used (SAGA 32 + and REFA 64, Twente Medical Systems International BV, the Netherlands; SD LTM 23 PLUS, Micromed S.p.A., Italy) with sampling frequencies of 256, 500, 1024 or 4000 Hz. Electrode impedances were kept below 20 kΩ.

*Preprocessing of EEG data* From the EEG recordings, we kept 19 channels (Fp1, Fp2,F7, F3, Fz, F4, F8, C3, Cz, C4, T7, T8, P7, P3, Pz, P4, P8, O1, O2). We re-referenced EEGs offline to the average of the vertex channel ((Fz + Cz)/2). Recordings were resampled to 256 Hz or to 250 Hz. EEGs were filtered offline using a Hamming windowed FIR band-pass filter between 0.2–35 Hz.

*ERPs analysis* Data analysis was performed with Matlab and the freely available EEGlab toolbox (version 2021.0),^40^ using ERPLAB 8.10 plugin.^41^ Preprocessed EEG data was epoched 200 ms prior and 1000 ms after the trigger events (i.e. target stimulus appearance), with baseline correction. We subsequently applied both a manual and automatic artefact rejection to discard the noisy trials, marking epochs containing peak to peak activity greater than 100 µV, within a moving window (interval −200 to 800 ms, width: 200 ms; steps: 50 ms). Trials were excluded if RT was longer than 1700 ms, shorter than 200 ms or if no fixation to any of the AOIs was made (see Supplemental Material S5 for our fixation related potential detection). A late ERP component was measured from central line channels (i.e. Fz, Cz, Pz).^27,28,42^ We averaged the EEG data sets per channel and computed the Grand Average for each group. Here, we quantified the mean amplitudes and latencies of peak amplitude from the extracted component for channel Pz only, since channel Fz and Cz were influenced by the choice of the common reference used.^43^ The code of all the analysis is freely available upon request.

### Clinical Data: Controls and Patients

Six healthy controls (25–29 years, 3 females) and 13 neurological patients with either epilepsy or TBI were included. Patients with TBI were assessed in the acute (A) phase (*≤* 24 h from injury), subacute (S) phase (48 h up to 3 months from injury) or both (A + S). Patient characteristics are summarized in Table 1. All patients were referred either to the epilepsy clinic Kempenhaeghe or to the Medisch Spectrum Twente for clinical evaluation. Local ethical approval was obtained in both institutions. Participants and their tutors (in case of participants younger than 16 years old) provided informed written consent, according to the approved research protocols.

**Table 1.** Demographic Characteristics of the Neurological Patients.

NC	Age	Sex	CT/MRI scan					Medication
GGEA	16	M	–					LEV
GGEA	13	F	–					ETX, Clobazam
GGEA	16	M	–					–
FE	26	M	–					CBZ, Clobazam, Fycompa
FE	26	F	right T vessel defect					–
TBI-A	25	F	SAH					–
TBI-S	75	F	normal					Clopidogrel, Omeprazol
TBI-S	18	F	normal					–
TBI-A + S	74	M	F-P bilateral SDH	SAH,	minor	F-P	right	HCTZ
TBI-A + S	59	M	normal					Tamsulosine
TBI-S	52	F	normal					Enalapril
TBI-A	62	M	F-P left SDH					–
TBI-S	53	M	normal					–

NC: neurological condition; GGEA: Genetic generalized epilepsy with absences; FE: focal epilepsy; TBI: traumatic brain injury. A(+S): acute(+subacute) phase; M: male; F: female; F: frontal; FP: fronto-parietal; SAH: traumatic subarachnoid hemorrhage; SDH: subdural hematome; T: temporal; LEV: Levetiracetam; ETX: Ethosuximide; CBZ: Carbamazepine; HCTZ: Hydrochloro-thiazide.

### Statistics

We compared differences of all metrics between the four groups using the module *statistics* in Python 3.8. A two-sampled Mann-Whitney U test was used for fixation parameters (i.e. fixation duration and time spent on) and RT-subcomponents to compare the mean of each patient group to the healthy volunteers.

We used the Mann-Whitney U test because our sample size is smaller than 30 and some of the variables measured are not parametric. Spearman's correlation was used to compare the relationship between saccadic amplitude, saccadic duration and saccade mean velocity. For the ERPs we used a two-sampled Mann-Whitney U test to compare differences between patients and controls. If p*<*0.05 we considered results statistically significant.

The code used for our analyses is available upon request from the corresponding author.

## Results

Our setup is suitable to measure features of attention both in controls and neurological patients in approximately 15 min within a clinical environment.

### Eye Movements

**Figure 2.**
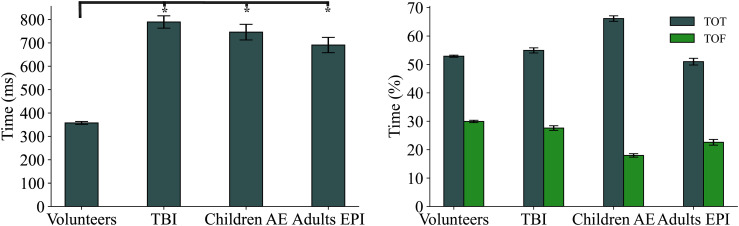
Left: total mean fixation duration for all groups; error bars show standard error of the mean. All patient groups differ from the healthy controls. TBI and children with AE show the largest durations. Asterisks represent p *<* 0*.*01. Right: Percentage of time included in a single trial of fixation spent on target (TOT) and on fixation cross (TOF). The only non-significant difference is observed in the TBI group, which shows a similar pattern of fixation on target to volunteers (p = 0.06, mean difference = 2%). Children with AE have a tendency to spend more time on the target compared to any other group (66% vs 52.9%, p 0*.*001), while they spend the least time on the fixation cross (18% vs 30%, p 0*.*001).

The overall mean fixation duration of the three neurological groups is significantly different from the average fixation duration of volunteers (p *<* 0.01), see Figure 2-left. Patients with TBI and children with AE show the largest fixation duration compared to healthy controls (mean difference = 431ms and 382 ms respectively).

Significant differences are also found in the amount of time (%) spent on AOIs, as shown in Figure 2-right. Time on target (TOT) and time on fixation cross (TOF) show the largest difference (13.2% and 11.9% respectively) in children with AE, while no significant differences are noted in TBI patients.

**Figure 3.**
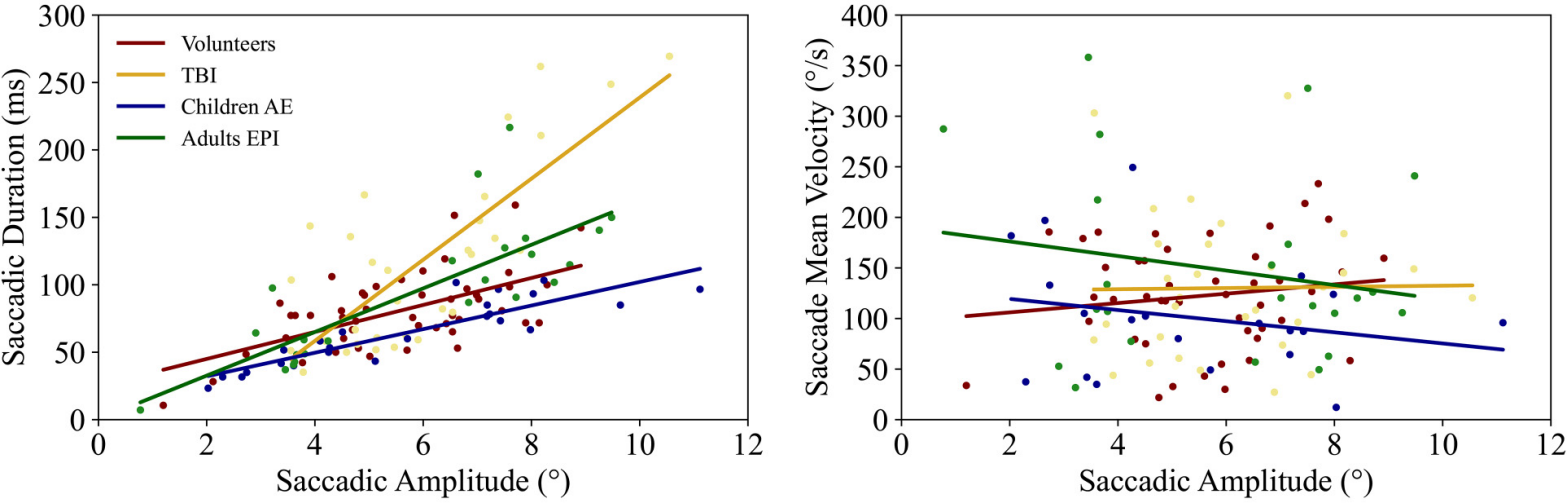
Left: saccadic durations and amplitudes are positively correlated for all the groups (p *< *0.05), children with AE show the strongest correlation (r = 0.88). TBI patients have overall longer saccades than the other three groups. Right: Saccadic mean velocity versus amplitude. No significant correlation was found for any of the groups included. Controls and TBI patients show a similar positive trend, while epilepsy patient reveal a negative trend.

We derived the so-called Main Sequence (i.e. relatively fixed relationships between the amplitude, duration and velocity of saccades) for each group, a standard metrics for the functioning of the visual nervous system.^44^ In Figure 3 we show the relationship between saccadic duration and mean velocity versus saccadic amplitude. Saccadic duration and amplitude are significantly correlated for all groups; no significant correlation was found for mean velocity and amplitude of saccades.

**Figure 4.**
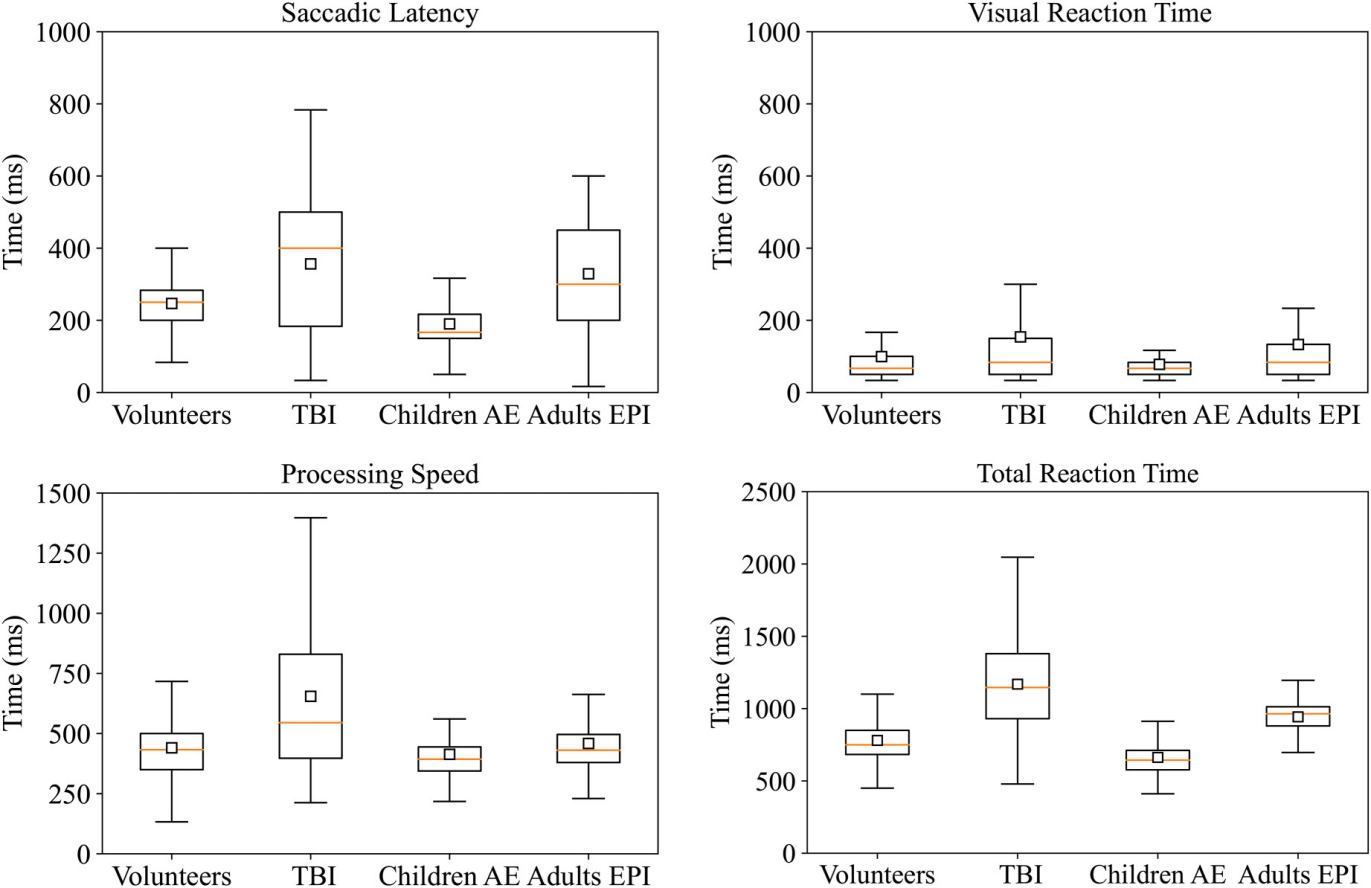
Reaction time subcomponents for all groups. Upper left: Saccadic Latencies differ between neurological patients and volunteers. All patient groups show statistically significant (p *<* 0.05) differences in SL, with a mean difference ranging from 56 ms in children with epilepsy to 109 ms for TBI patients. Upper right: Differences in Visual Reaction Time are significant for all our groups too, with a mean difference ranging from 21 ms to 54 ms. Lower left: Processing speed shows a significant difference for patients with TBI and children with AE (p *<* 0.05, mean difference = 214 ms and 26 ms), but not for adults with epilepsy. Lower right: Total RT differs from volunteers in all the patients’ groups, with a mean difference ranging from 106 ms to 396 ms.

Subcomponents of RT (i.e. SL, VRT, PS) extracted with the ET are summarized in Figure 4. Of note, variance within trials included is the largest for patients in the acute phase of TBI for all the obtained components. Total RT, SL and VRT differ for all the neurological groups compared to controls, while PS is not significantly different in adults with epilepsy.

### ERPs

**Figure 5.**
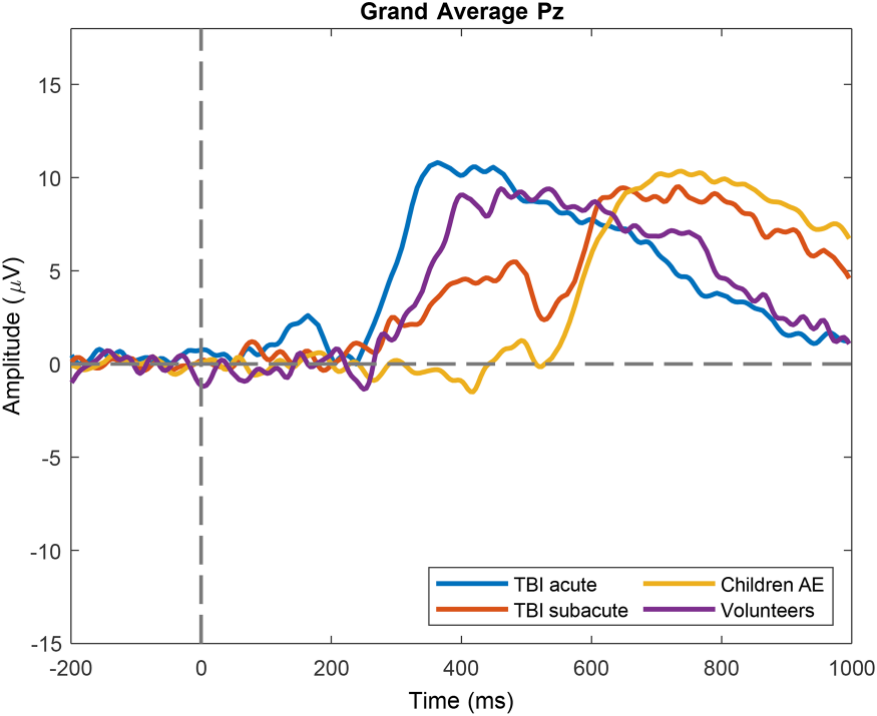
Grand average ERP for channel Pz. A late positive deflection is present, with onset at approximately 300–500 ms after stimulus up to 800–1000 ms. The latencies between the volunteer group and the TBI groups (both TBI-A and TBI-S) differ with approximately 250 ms. Visually, the amplitudes of the TBI-A and the TBI-S group differ but these differences were not statistically significant (p > 0.05).

The number of trials included per subject ranged from 7% to 93%. Our ERP analyses conducted on the centro-parietal channels of the EEG data revealed a clear late component for all the groups involved in this study, shown in Figure 5. Earlier components (e.g. N1, P1) could not be detected, possibly due to the low number of trials included.

In Table 2 we summarize the mean amplitude and peak latency per group of the Grand Averaged late positive potential we found. No statistically significant differences were found between neurological groups and controls.

**Table 2.** Mean Amplitude and Peak Latency for Window 250–700 ms, Channel Pz. Differences Between Controls and Patients Were Not Statistically Significant.

Group	Amplitude (± SEM) (µV)	Latency (± SEM) (ms)
TBI-A	4.8 (3.1)	648 (67)
TBI-S	2.2 (1.2)	492 (42)
CAE	8.0 (2.45)	363 (92)
Controls	6.7 (1.8)	461 (34)

TBI-A: TBI acute; TBI-S: TBI subacute; CAE: childhood absence epilepsy.

**Figure 6.**
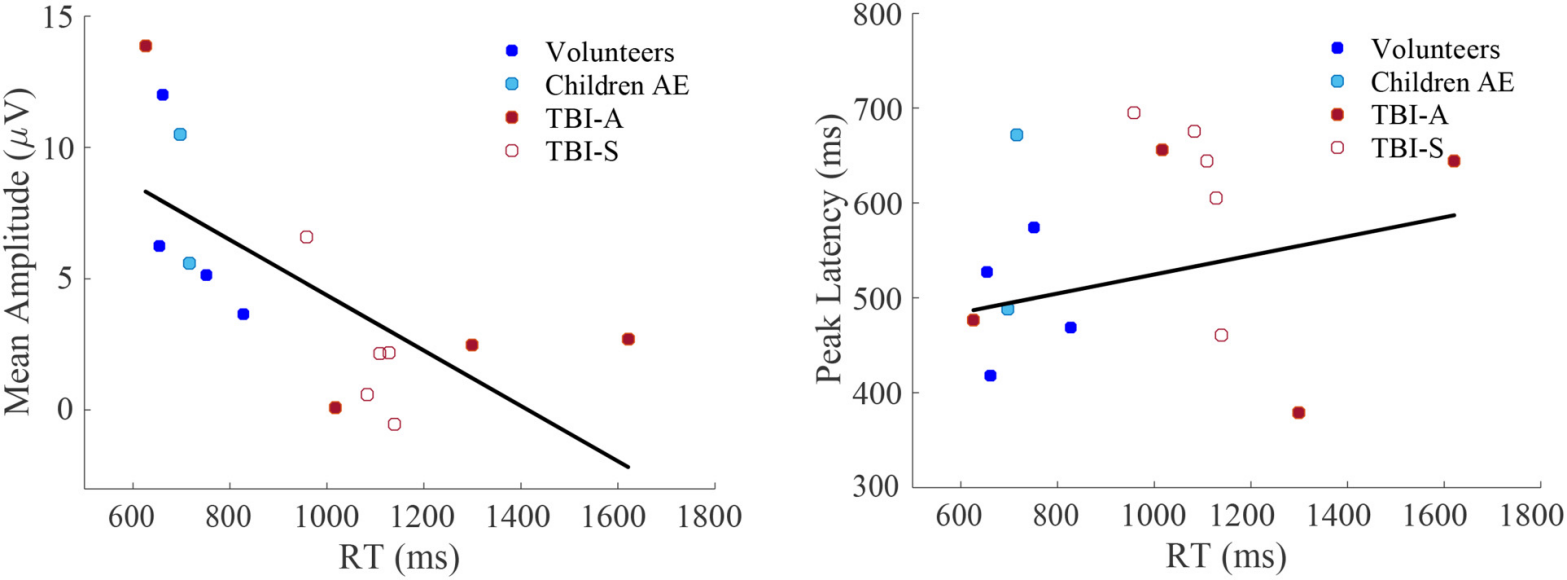
Mean amplitude and peak latencies as a function of reaction time for all data, using Spearman correlation. Left: A significant (p < 0.01, rho = −0.8) negative correlation between mean amplitude and RT is observed. Right: A non-significant (p > 0.05, rho = 0.1), slightly positive relation between peak latencies and RTs is present.

Finally, we evaluated correlations among RTs, late positive potential mean amplitude and peak latency for all subjects using Spearman's correlation coefficient, shown in Figure 6. Mean amplitudes and RTs are negatively correlated (p *<* 0.01, rho = −0.8), while peak latencies and RTs show a small positive, non-significant relationship (p > 0.05, rho = 0.1).

## Discussion

We present a multimodal test that allows a quick assessment of visual attention in clinical settings. We evaluated the potential of our test with pilot measurements in patients with neurological disorders, in particular epilepsy and mild TBI.

The features extracted with our test provide relevant metrics for attention, and can both enrich our understanding of how attention changes in these neurological conditions and allow monitoring of progression.

Since usually a high inter-subject variability exists in neurological patients, resulting from e.g. age, medication intake or etiology of the diseases, and different attention networks may be affected, a combination of multiple metrics adds value to the assessment of changes in attention. For instance, patients may show normal eye movements but abnormal ERPs.

### Eye Movements

Particular characteristics from eye movements reflect cognitive impairment in groups with brain lesions or neurological disorders.^18,26,33,45^ Our ET results show that our setup has potential to discriminate between healthy subjects and patients with neurological disorders. Both fixation duration and percentage of time spent on target AOIs exhibit different patterns in neurological disorders compared to volunteers (*cf* Figure 2). Specifically, TBI patients show the greatest increase in fixation duration, significantly longer than controls. Our finding is in accordance with a recent review,^20^ which indicates fixation duration as one of the eye metrics altered in mTBI compared to controls. We also find that pediatric patients with absence epilepsy show an increase in fixation duration and time spent on AOI compared to controls. This is at variance with the only ET study on epilepsy and fixation duration we found, where epileptic children show shorter fixation duration on visual target stimuli compared to controls.^21^ This discrepancy may result from the type of epilepsy studied: our three pediatric patients were all diagnosed with absence epilepsy, a syndrome well known for its “staring patterns” as a behavioral manifestation of seizures,^46^ while Hunter et al included children with early onset epilepsy, mixing different epilepsy syndromes. One of our patient experienced an absence seizure during the task, and possibly interictal epileptiform discharges (IED) were included in the trials we analysed. An increase of fixation time is, therefore, expected in AE. Other possible reasons for our discrepant findings derive from our unmatched control group, the medication used by these patients and the small number of subjects included.

The results of our investigation of the Main Sequence revealed that duration and amplitude of saccades are positively correlated for the groups we examined (Figure 3-left). The TBI patients in the acute phase show the most dissimilar trend, due to longer saccadic durations, which is in line with previous findings.^18^ Amplitude and mean velocity, instead, do not show a significant correlation in our subjects (Figure 3-right). The TBI group in the acute phase shows a similar positive trend comparable to controls, while epilepsy patients manifest an opposite trend. The literature on this topic is scarce, but one study to date^47^ shows that the saccadic Main Sequence is similar to controls in patients in the chronic phase of TBI,^47^ even though no evidence has been collected in the acute and sub-acute phase. Here we demonstrate that the Main Sequence can be extrapolated with our set-up, even if more patients are needed to determine possible significant differences between TBI, epilepsy patients and controls.

### ERPs

ERPs have been used extensively to detect modular cognitive features and impairment in the time domain. Late ERP components—e.g. the P300, late positive potential—are involved in high-level cognition, such as orientation of attention, stimulus evaluation or categorization and memory recognition. In particular, the late positive potential may represent processes that contribute to the representation of recollected information^48^ or orienting of attention to recollected information.^49^ In healthy subjects, the latency of P300 ranges between 250–500 ms, and it varies with age.^50^

Our ERP analyses shows that our test is able to provide such late ERP components. We focused specifically on late components of channel Pz. The component we found may also relate—at least partially- to P300. P300 has been extensively used to determine cognitive decline and its association with epilepsy, where its latency is significantly prolonged in epilepsy.^28^

For the ERP analysis, we could only include two pediatric patients with AE. The ERP latencies of these two patients were similar to controls (mean difference = 83 ms), and their mean amplitude slightly differed from controls (mean difference = 1.28 µV), but this was not significant.

TBI patients tend to show a longer latency in the acute phase, while latencies in the subacute phase were reported to be similar to controls.^18^ We found that both TBI- A and TBI-S seem to have a longer late positive potential latency than controls, but no statistically significant differences were detected. In fact, the increase of peak latency may be the result of the differences in age, as the peak latency increases with age.^50^

The small number of participants and the variable amount of trials included in our ERP analysis can influence our results. Nevertheless, our focus here is to illustrate that our novel measurement tool allows reliable measurement of ERPs even with a small sample. More extensive ERP analysis can be conducted using e.g. more electrodes, focusing on the laterality and topological differences of the components detected.

### Combined Setup

Measuring reaction time (RT) can be easy with simple behavioral tasks. Nevertheless, the composite nature of recognizing, processing and acting after an external stimulus needs a more in-depth analysis of the subcomponents of RT. Total RT measurements combined with gaze detection by an eye tracker allow subdivision of the RT into three components:

i) the SL, the time from stimulus onset to the start of the saccade towards the target stimulus; ii) the VRT, the time from the start of the saccade to the gaze reaching of target stimulus and iii) the PS, the time from gaze reaching of target stimulus to button press. Subdividing RT adds valuable information about the contribution of different cognitive processes underlying standard RT. SL is a measure of afferent visual conduction, providing an evaluation of the state of the peripheral vision.^51^ VRT and PS represents low level processes of visual stimuli and higher level elaboration of stimuli and decision making respectively.^52^ Only one study to date compared the three subcomponents of RT in neurological disorders,^33^ where PS is longer than the other two components in patients with TBI compared to controls, while SL and VRT are significantly shorter. Our findings show that SL, VRT and total RT differ significantly for all our neurological patients group as compared to controls (Figure 4), while PS differs only in TBI and CAE. Our results are partially in line with Lange et al.^33^: we found significantly longer RTs and PS in TBI, but shorter SL. A main discrepancy derives from the type of TBI investigated, which in our case is mild but it is not specified in the study by Lange and colleagues. Although not included in our study, RT components as modulated by trial condition (uncued vs valid cue vs invalid cue) could also be used to determine the ability of the system to measure different attentional processes.

With our set up we were able to evaluate possible association between RTs and ERP metrics (i.e. mean amplitude and peak latencies), too. While our current sample cannot show significant differences among groups, a significant negative relation between RT and ERP mean amplitude was found (Figure 6). This association may be useful to determine the impairment of attention of neurological patients.

Our study has several limitations. First, our sample of controls and neurological patients is small, which may impact our results. Future work focusing on more complete analyses should include a larger number of subjects. Second, healthy controls were not age and sex matched. Further, several patients used medication that may affect visual attention. Despite these limitations, we show that it is feasible to use our test in a clinical setting and to extract parameters of visual attention in patients with epilepsy or TBI. Of note, data quality and the rate of successful test administrations are satisfactory and allow the extrapolation of the presented variables. Moreover, all patients found it pleasant to complete the task. Further analyses may be applied in the future on the rich dataset extracted from this test, using a larger cohort of patients and controls. For example evaluation of EEG power spectrum or of EEG components (e.g. via ICA), more extensive ERP analysis, or in-depth multimodal testing using e.g. machine learning can be employed to additionally investigate the construct of visual attention.

## Conclusion

We show that our test possibly allows fast clinical assessment of both singular and combined markers of visual attention in patients with neurological disorders. These have potential to assist in personalized medicine and track improvement.

## Supplemental Material

sj-docx-1-eeg-10.1177_15500594221129962 - Supplemental material for A Potential Multimodal Test for Clinical Assessment of Visual Attention in Neurological Disorders Click here for additional data file. Supplemental material, sj-docx-1-eeg-10.1177_15500594221129962 for A Potential Multimodal Test for Clinical Assessment of Visual Attention in Neurological Disorders by Valentina Barone, Johannes P. van Dijk, Mariette H.J.A. Debeij-van Hall and Michel J.A.M. van Putten in Clinical EEG and Neuroscience
