# Supplementary material for: A Potential Multimodal Test for Clinical Assessment of Visual Attention in Neurological Disorders
Source: Clin EEG Neurosci. 2022 Oct 3;54(5):512–21. doi: 10.1177/15500594221129962 (PMC10411032; doi:10.1177/15500594221129962)
Supplement: sj-docx-1-eeg-10.1177_15500594221129962 - Supplemental material for A Potential Multimodal Test for Clinical Assessment of Visual Attention in Neurological Disorders [file sj-docx-1-eeg-10.1177_15500594221129962.docx]

A potential multimodal test for clinical assessment of visual attention in neurological disorders

Supplementary Material

# S1 Task sequence

The task sequence is shown in Figure S1.


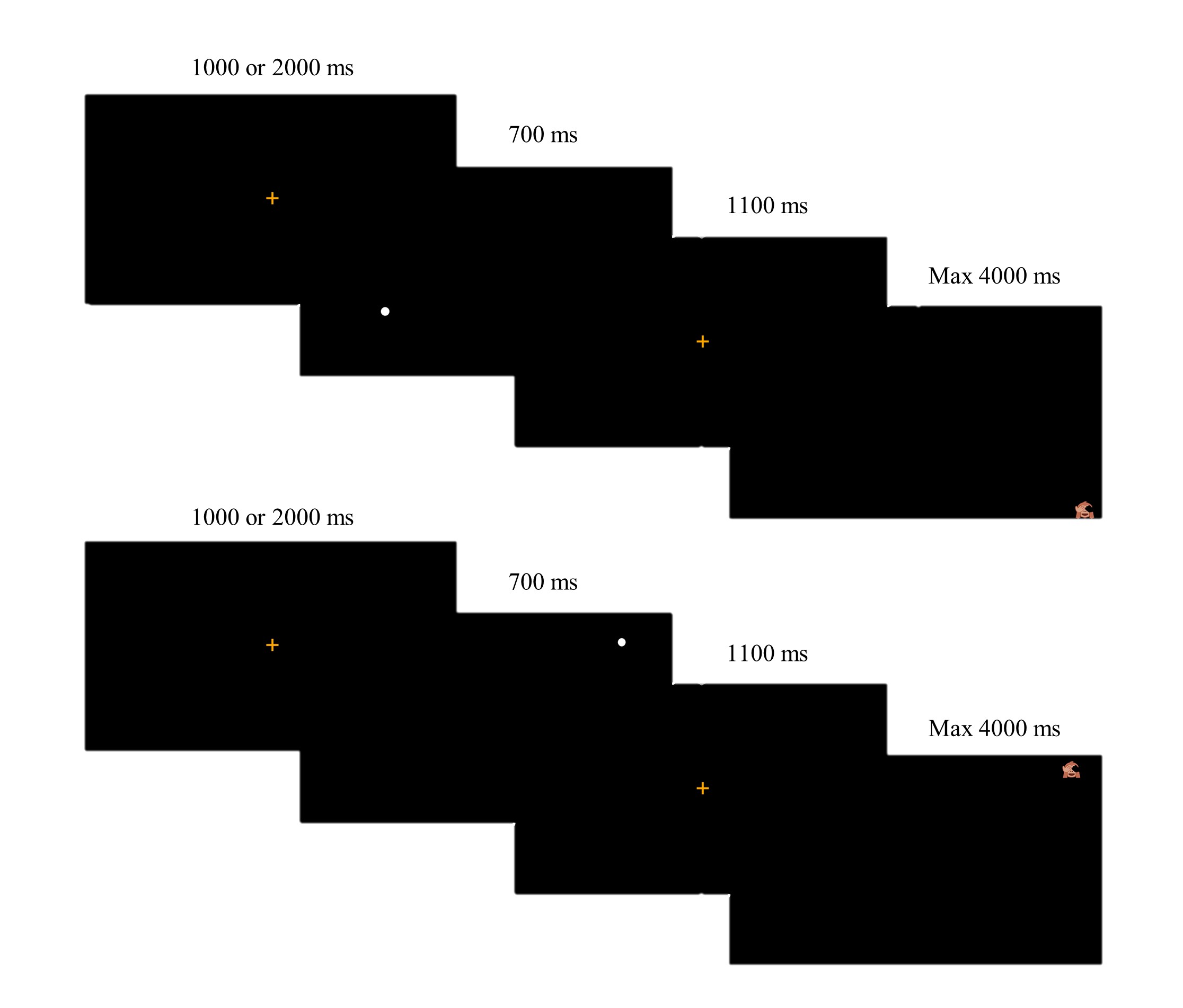


Figure 1. Sequence and timing of stimuli presentation during the CRT task. The fixation cross (30 x 30 height of the window) in the centre of the screen is followed by a cue (a white dot) which can be incongruent (top figure) or congruent (bottom figure) to the position of the target stimulus. After the cue, the fixation cross is presented for 1.1 s, before the target stimulus (monkey face) appears either in the position of the cue (top figure) or not (bottom figure). There are 8 possible positions for the target and the cue. The left or right eye of the monkey face can be covered by a black hand, which should result in a left or right button press by the subject.

# S2 Time synchronization

Given the brevity of the events we want to record and compare using different measurement techniques - i.e. in the order of ms - we must carefully take into consideration the time synchronization of EEG, ET and the CRT game. The CRT task and the ET are synchronized using Tobii Pro SDK and PyGaze, an open-source package used in Python 3.7.6. The clock of the eye tracker is aligned to the clock used by PyGaze in OS, therefore the two can be synchronized with no delay. The EEG is synchronized with the game via a TTL trigger event. A trigger is sent to the EEG amplifier with a general-purpose input/output (compatible GPIO, 5V DC relays, Numato Lab) when a target stimulus appears. The delay of the trigger reaches a maximum of 3 ms, and is, thus, negligible for our analyses.

Furthermore, we considered the monitor delay, that is the lag between the software command to display a stimulus and the actual appearance of the stimulus on the monitor. A delay of 15-18 ms between two monitor displays was found, based on the monitor refresh rate (59 Hz). This delay does not impact the synchronization of the game with the EEG and ET, since the clock of the trigger command is independent from the clock of stimuli display. The delay in the functions execution between the trigger command and the stimulus appearance is ≈ 0.1 ms, and is therefore, negligible.

# S3 Eye movements detection

We analysed the ET data comparing two different detection algorithms: a dispersion-based detection (I-DT) and a velocity-based detection (I-VT). This choice was motivated by the type of data available. We could only retrieve gaze positions data in pixels from our ET log file, therefore the derivation of visual angles is subject to the assumption of a constant distance of the subject from the screen. The distance of the participant from the screen cannot be defined accurately after the calibration procedure. We investigated the degree of influence of the method chosen on the extrapolation of eye features before proceeding further with our analyses. Specifically, we determined if our detection of RT subcomponents and relationship between saccadic parameters is comparable using the two algorithms.

## S3.1 Dispersion-based detection I-DT

Our I-DT is a pointwise calculation of eye movements parameters. The main assumption of this algorithm is that fixation points tend to cluster together. Therefore groups of consecutive points within a particular dispersion are identified as fixations.

## S3.2 Fixations

We divide the gaze data according to the number of trials. Each trial is defined between the appearance of the target stimulus and its disappearing, due either to a button press or a time-out. For every trial we define two areas of interest: the fixation cross, *T*_X_ and the target stimulus, *T*.

The coordinates in pixels of *T*_stim_ is determined from the OS log file. We adjust the coordinates in pixels from OS to the coordinates of Tobii (xOS + 960, 540 - yOS). From these coordinates, we create a square, with the x,y coordinates as its centre (side = 200 pixels). We then derive the circumference in which the square is inscribed (radius = (side* **√**2 )/2). The resulting circumference is our *T*_stim_. The same procedure is applied to identify *T*_X_ (same for each trial), for which we use the coordinates (0 +960, 540-0). For each trial, we average the gaze points between the left and the right eye for both the x and the y coordinates. We check visually the gaze points of each subject, as shown in Fig. S2. If the movements of one of the two eye result in gaze points≥100 pixels compared to the ”correct” gaze positions of the *T*_X_ or *T*_stim_, we use the gaze positions of the correct eye only. We, then, check if the averaged gaze points are contained in the *T*_stim_. The time spent on the *T*_stim_ for each trial is detected as a percentage of the total duration of the trial. With the same procedure we extrapolate the time spent on the fixation cross, before the eye starts to move towards the target stimulus. Fixations are considered as such only when the time spent on either the *T*_stim_ or *T*_X_ is larger than 10 samples (i.e. at least 160.6 ms).

## S3.3 Saccades

A saccade is detected if the number of samples between the fixation on the *T*_X_ and the fixation on the *T*_stim_ is ≥ 4 samples. We determine the beginning of the saccade as the first sample outside the *T*_X_ in the time period immediately preceding the first fixation on the *T*_stim_. We define the ending of the saccade as the first sample point on the *T*_stim_. The amplitude of the


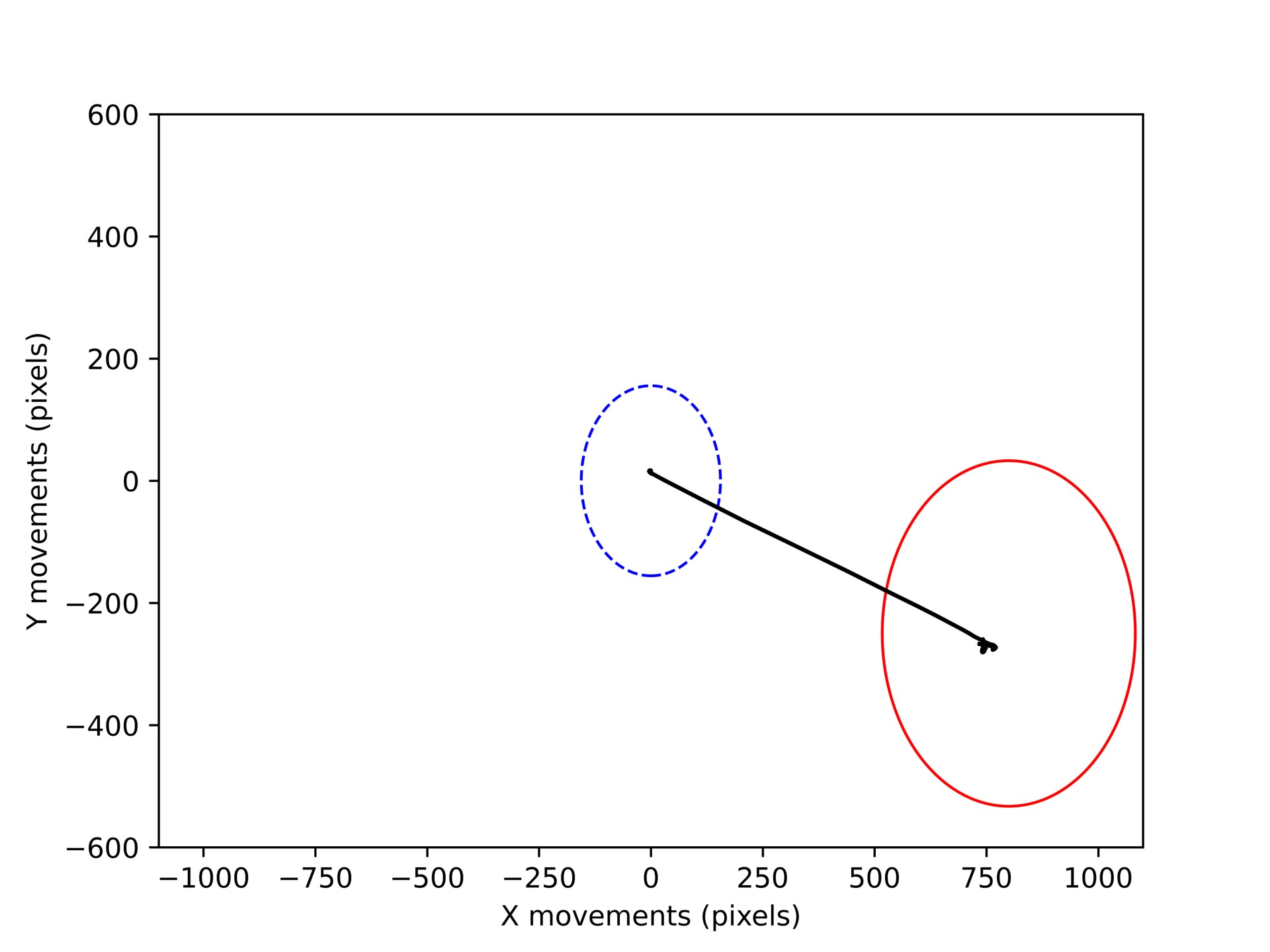


Figure 2. Visual inspection of gaze movement per single trial, including *T*_X_ (in blue) and *T*_stim_ (in red).

saccade is defined as

*S* = **√***x*^2^ + *y*^2^*.* (1)

with x being the x coordinates in pixel of the gaze data found at end of the saccade subtracted by the x coordinates of the beginning of the saccade, and y being the y coordinates in pixel of the end of the saccade subtracted by the y coordinates of the beginning of the saccade. We are aware that within the begin and end points of our saccades, other sub-saccades may be present, but the detection of these is out of our scope. In fact, our main goal is to divide our trials into three specific moments included in the total RT (i.e. SL, VRT, PS). Therefore, we consider the entire time window from *T*_X_ to *T*_stim_ as a single saccadic movement.

## S3.4 Reaction Times

RTs represent the total time between the appearance of the target stimulus on the screen up until the end of the trial (i.e. when stimulus disappears either for a button press or time-out). The extrapolation of subcomponents of RT has been applied as follows: i) Saccadic Latency is the time in milliseconds derived from the first sample defined as a saccadic movement; ii) Visual Reaction Time is defined as the total length of the first saccade from the fixation cross to the target stimulus; iii) Processing Speed is the subtraction of VRT and SL from the total RT. If one of the three RT components could not be detected we assign a value of zero to the three components, discarding the entire trial. We calculate the percentage of these components as part of the total RT.

## S3.5 Velocity-based detection I-VT

To obtain information about the velocity of saccades, we used a velocitythreshold based algorithm. In order to transform our pixel data into degrees of visual angle we need to establish a few parameters: the monitor height (h = 19.5 cm), a constant distance from the screen (d = 60 cm) and the vertical resolution of the monitor in pixels (r = 1080 pixels). We transform pixels to degrees of visual angle, *α_v_* as follows:


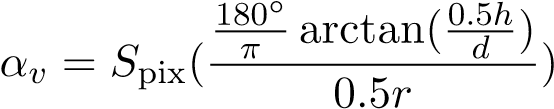
*,* (2)

where *S*_pix_ is the size in pixel of our gaze positions, *h* is the height of the screen in cm, *r* the vertical resolution of the monitor in pixels and *d* is the distance from the screen in cm.

## S3.6 Saccades

If both *T*_X_ and *T*_stim_ have been fixated, we calculate the angular velocity between each pixel point on the screen, starting from the first point laying outside *T*_X_. If the angular velocity is larger than 35^◦^/s (point-to-point velocity threshold partly based on I-VT detection with Tobii systems [1]), we define the beginning of a saccade. The end of the saccade is the first sample in which the eyes reach the target stimulus. Saccade amplitude is the angle between *T*_X_ and the first point in *T*_stim_. We obtain the saccadic mean velocity dividing the amplitude by the saccade duration. The gain of our saccade is the ratio of the saccade amplitude divided by the size in degree of *T*_stim_ steps.

## S3.7 Reaction Times

RT and its subcomponents are defined as described for the I-DT algorithm, using saccades and fixations detected via the I-VT algorithm instead.

## S3.8 Comparing the two algorithms

We compare the results of our I-DT and I-VT detection of saccades. Both algorithms are affected by the sampling frequency of our ET (fs = 60 Hz).

Indeed, taken singularly, each duration we derive (both for fixations and saccades) is subject to the sampling time (i.e. 16.6 ms). A way to reduce this ”noise” in both detection methods is to average our results per each target position of our *T*_stim_ per subject. As shown in Fig. S3, both the relationship between saccadic duration and amplitude (i.e. Main Sequence) and the extrapolation of RT subcomponents is comparable between our I-VT and I-DT algorithms.


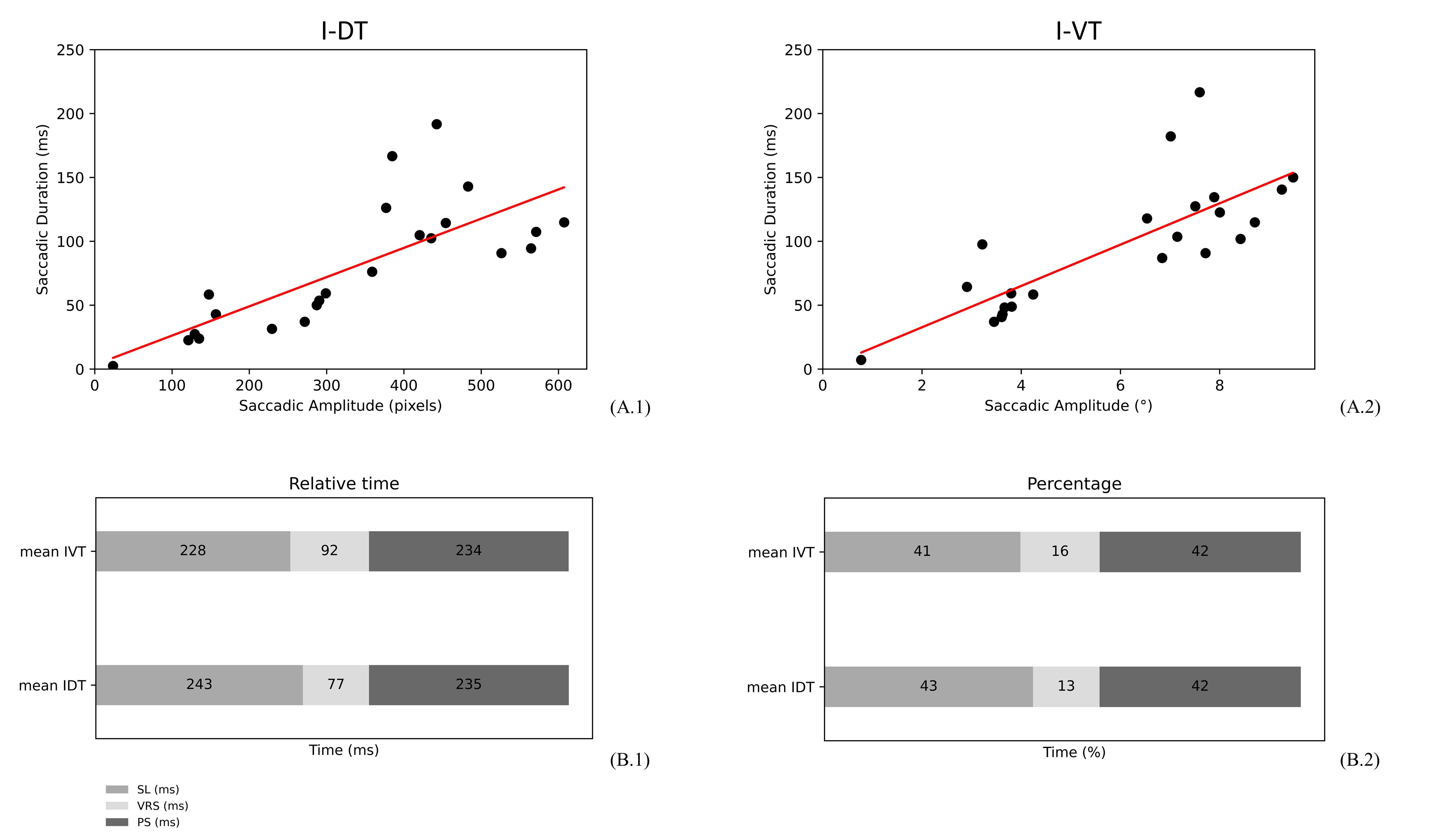


Figure 3. Comparison of I-DT and I-VT detection of saccade parameters and RT subcomponents for adult epilepsy patients. (A) Both the upper plots show the expected fixed relationship between saccade duration and amplitude. (B) RT subcomponents detected as relative time in ms (B.1) and percentage (B.2) of the total RT.

The distance value in cm used to derive the visual angle in degrees will impact the absolute value of the amplitude and velocity parameters of our saccades, but it does not impact the overall detection of the RT components we are interested in. In Fig. S4 we compare the extrapolation of SL and VRT using five different distances (i.e. 40, 60, 70, 90 and 110 cm) for one subject. The estimate shows that the averages of SLs and VRTs in the range of 40 and 70 cm do not change. On the other hand, a distance bigger than 90 cm presents a small difference in the averaged values (max 3 ms) both for the detection of SL (Fig. S4-left) and VRT (Fig. S4-right). This deviation is negligible for the scope of this paper. For this subject, the number of trials with different SL and VRT values according to the distance chosen lie in the range of 1-4 trials. PS values are the same for every distance chosen, since both SL and VRT change following the same decreasing pattern.


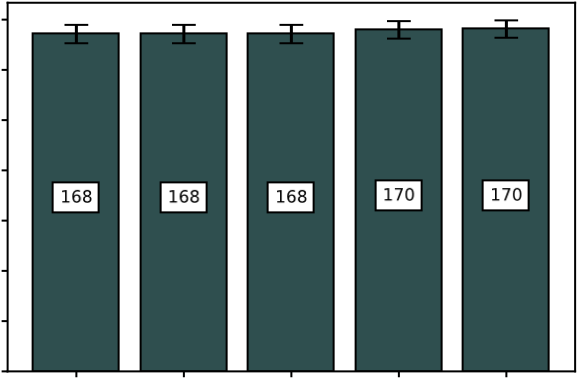

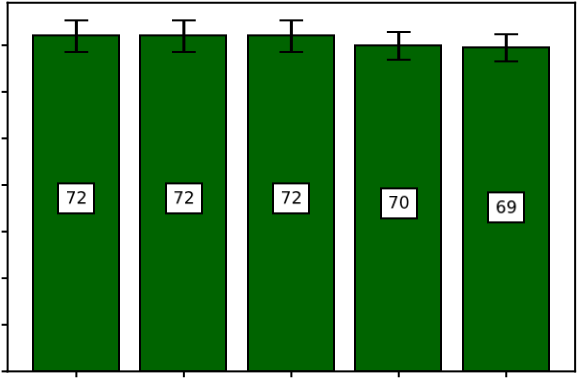


Figure 4. I-VT detection of averaged SL and VRT with different given distances for all trials (N = 120) for one patient with CAE. Five different distances are included (40, 60, 70, 90 and 110 cm).

# S4 Saccadic Gain

We define saccadic gain, *S*_gain_ as *S*_gain_ = *S*_amp/_ *T*_size_, where *T*_size_ is the size in degrees of the *T*_stim_. We used a two-sampled MannWhitney U test for saccadic gain to compare the mean of each patient group to the healthy volunteers. In our pilot results the saccadic gain differs between volunteers and epilepsy patients (p *<*0.05), while it is similar in patients in the acute phase of TBI (p= 0.08, two-tailed). This is not in line with our expectation of patients with TBI showing a deviated pattern of saccadic gain.

Figure 5. Saccadic gain is the ratio of the saccade amplitude divided by the size in degree of *T*_stim_. Gains of *<*1 indicate the saccade was too small or hypometric, while gains of *>*1 indicate the saccade was too large or hypermetric.

# S5 Main Sequence


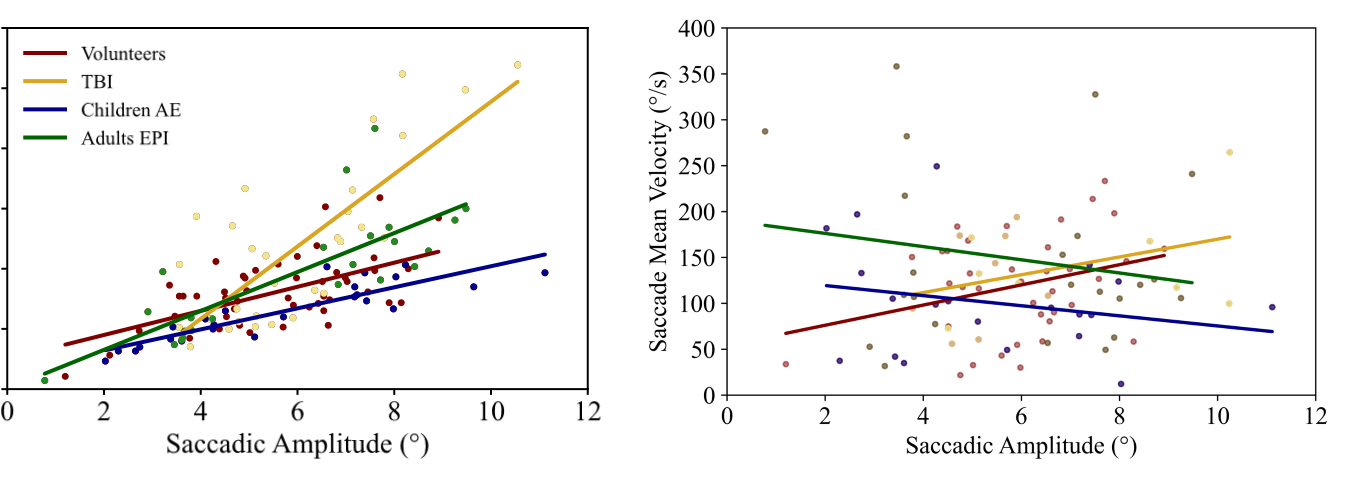


Figure 6**.** Left: Saccadic durations and amplitudes are positively correlated for all the groups (p *<*0.05), children with AE show the strongest correlation (r = 0.88). TBI patients have overall longer saccades than the other three groups. Right: Saccadic mean velocity vs amplitude. No significant correlation was found for any of the groups included. Controls and TBI patients show a similar positive trend, while epilepsy patient reveal a negative trend.

# References

1. A. Olsen, R. Matos, Identifying parameter values for an I-VT fixation filter suitable for handling data sampled with various sampling frequencies, Eye Tracking Research and Applications Symposium (ETRA) 1 (212) (2012) 317–320.
2. A. Buonocore, O. Dimigen, D. Melcher, Post-saccadic face processing is modulated by pre-saccadic preview: Evidence from fixation-related potentials, Journal of Neuroscience 40 (10) (2020) 2305–2313.
3. J. Kamienkowski, M. Ison, M. Quiroga, Fixation-related potentials in visual search : A combined EEG and eye tracking study 12 (2012) 1–20.
4. C. Korner, V. Braunstein, M. Stangl, A. Schl¨ ogl, C. Neuper, A. Ischebeck,¨ Sequential effects in continued visual search: using fixation-related potentials to compare distractor processing before and after target detection, Psychophysiology 51 (4) (2014) 385–95.
